# Complete hydatidiform mole with coexisting fetus: A case report

**DOI:** 10.1016/j.crwh.2025.e00770

**Published:** 2025-11-29

**Authors:** Yuanhua Xiang, Wei Zhang, Dongmin Chen, Ting Luo

**Affiliations:** Department of Ultrasound Medicine, Taizhou Hospital of Zhejiang Province Affiliated With Wenzhou Medical University, Taizhou 317000, Zhejiang, China

**Keywords:** Complete hydatidiform mole, Coexisting fetus, Gestational trophoblastic neoplasia, Prenatal diagnosis, β-human chorionic gonadotropin

## Abstract

Complete hydatidiform mole with coexisting fetus (CHMCF) is an exceptionally rare and clinically challenging obstetric condition. Its diagnosis and management require a high index of suspicion and a multidisciplinary approach. This report presents the case of a woman in her 30s who presented for a routine prenatal examination at 15 weeks of gestation. Ultrasonography revealed a placenta with a mixed echogenicity area exhibiting a characteristic honeycomb-like appearance, alongside a coexisting viable fetus with normal biometry. Serum β-human chorionic gonadotropin (β-hCG) was markedly elevated at 259,762 mIU/mL. Through serial monitoring and multidisciplinary consultation, a diagnosis of CHMCF was suspected and later confirmed by postnatal genetic analysis. The pregnancy was complicated by persistent vaginal bleeding at 21 weeks, leading to termination following a comprehensive risk-benefit assessment. This case underscores the diagnostic challenges, the critical role of cytogenetic analysis, the importance of patient counseling regarding the significantly elevated risks of obstetric complications and gestational trophoblastic neoplasia (GTN), and the necessity for meticulous post-evacuation surveillance. This report aims to enhance clinical awareness and outline a structured management protocol for this rare entity.

## Highlights

• Diagnosis requires ultrasound showing honeycomb placenta with coexisting fetus, elevated β-hCG, and histopathological confirmation.
• Cytogenetic analysis is often required for definitive confirmation of the diagnosis.
• Management requires balancing maternal risks (hemorrhage, malignancy) with fetal viability through multidisciplinary counseling.
• Strict β-hCG monitoring and contraception for at least 6 months post-evacuation are essential to detect persistent GTN.

## 1 Introduction

Complete hydatidiform mole with coexisting fetus (CHMCF) represents an exceptionally rare obstetric condition, with an estimated incidence of 1 in 22,000 to 100,000 pregnancies [1]. This condition differs from partial hydatidiform mole (PHM) in that it involves a diploid molar tissue coexisting with a chromosomally normal fetus, typically arising from dizygotic twin conception where one conceptus undergoes complete molar transformation [2]. The increasing utilization of assisted reproductive technologies (ART) and ovulation induction has contributed to a progressive rise in CHMCF incidence in recent years [3]. The condition presents unique diagnostic and management challenges, as clinicians must balance the potential for fetal survival against significant maternal risks, including severe preeclampsia, hyperthyroidism, hemorrhage, and, most critically, the development of gestational trophoblastic neoplasia (GTN) [4].

While singleton partial hydatidiform mole carries a GTN risk of 15–20 %, CHMCF pregnancies demonstrate a substantially elevated risk, ranging from 27 % to 46 %. Additionally, these pregnancies are associated with high rates of obstetric complications, including first-trimester miscarriage (40 %) and preterm delivery (36 %) [5]. Despite advances in prenatal imaging and molecular diagnostics, CHMCF remains diagnostically challenging, often requiring invasive prenatal testing for definitive differentiation from other placental abnormalities such as placental mesenchymal dysplasia [6].

The present report concerns a case of CHMCF diagnosed in the second trimester. It illustrates the characteristic ultrasound findings, laboratory parameters, and the complex decision-making process involved in management. It contributes to the limited literature on CHMCF and emphasizes the critical importance of multidisciplinary consultation, thorough patient counseling, and vigilant postpartum surveillance in optimizing maternal outcomes.

## 2 Case Presentation

A woman in her early 30s presented to the obstetrics clinic for routine prenatal examination at 15 weeks of gestation. The pregnancy was spontaneous, and the patient had an unremarkable obstetric history, with one previous uncomplicated term vaginal delivery. She denied any history of gestational trophoblastic disease, recurrent miscarriage, or family history of molar pregnancy.

**Fig. 1:**
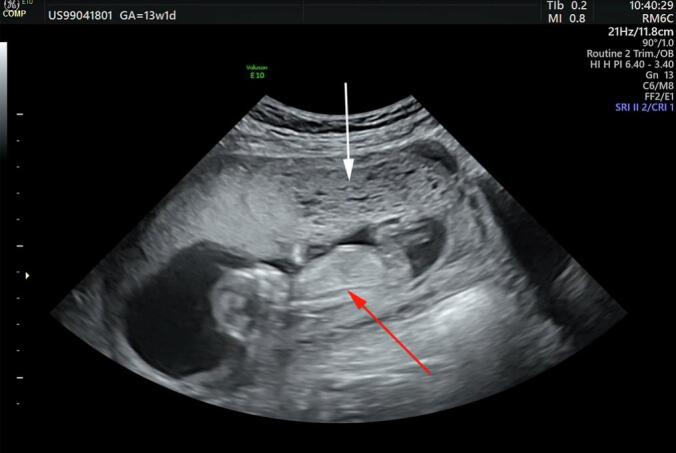
Ultrasound findings at 15 weeks of gestation demonstrating normal fetal parts (red arrow) alongside molar placenta (white arrow). (For interpretation of the references to colour in this figure legend, the reader is referred to the web version of this article.)

Obstetric ultrasonography performed at 15 weeks raised several concerns. A mixed echogenicity area was identified within the placenta, exhibiting a characteristic honeycomb-like or “Swiss cheese” appearance. Notably, a coexisting viable fetus with appropriate measurements for gestational age and normal morphology was visualized (Fig. 1). Fetal cardiac activity was present with a normal heart rate. No structural anomalies were detected on detailed fetal survey.

Given the suspicious placental findings, serum β-human chorionic gonadotropin (β-hCG) testing reported a concentration of 259,762 mIU/mL—significantly higher than expected for gestational age. Thyroid function tests and complete blood count were within normal limits. The combination of characteristic ultrasound findings and grossly elevated β-hCG levels raised strong suspicion for CHMCF. The patient was counseled extensively regarding the prognosis, maternal risks, and management options. She expressed a strong desire for pregnancy continuation and was managed with expectant care under close surveillance.

At 21 weeks of gestation, the patient developed persistent vaginal bleeding. Conservative management was initiated, including bed rest and the administration of intravenous tranexamic acid (1 g once daily), combined with progesterone supplementation (dydrogesterone 10 mg orally twice daily). However, the bleeding persisted and progressively worsened. The fetus remained viable with normal biometric parameters; however, given the uncontrolled bleeding and associated maternal risks, continuation of pregnancy became untenable.

**Fig. 2:**
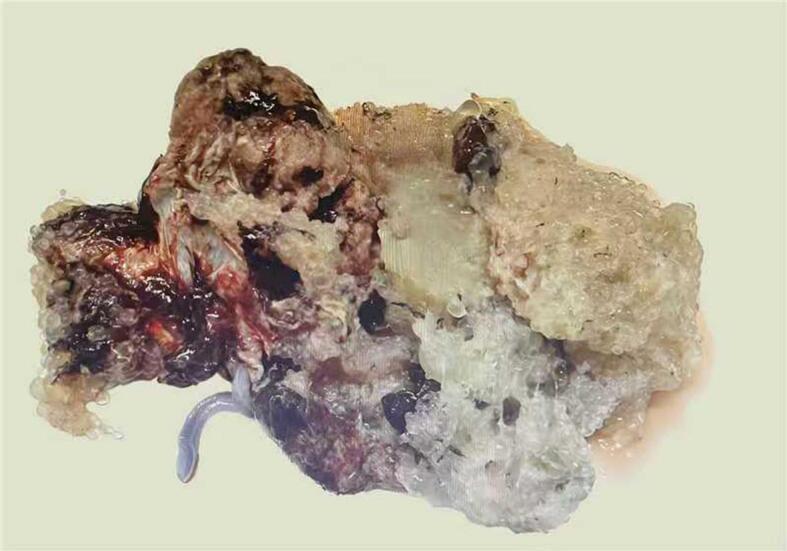
The morphological appearance of the placenta.

Following a repeat multidisciplinary consultation and a comprehensive reassessment of the escalating maternal risks versus the minimal chance of a viable neonatal outcome, the difficult decision was made to terminate the pregnancy. Given the unique challenges of CHMCF, a combined medical-surgical approach was employed. Medical induction was initiated with mifepristone 200 mg orally for cervical priming, followed 24–48 h later by misoprostol 400 μg vaginally every 3–4 h. Following fetal expulsion, surgical evacuation was performed under general anesthesia using ultrasound-guided suction curettage to ensure complete removal of the extensive molar tissue (Fig. 2).

**Fig. 3:**
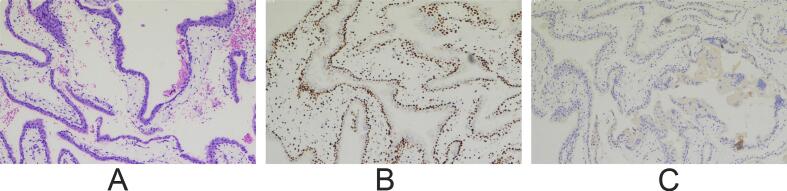
Pathology of the patient's placenta. (A) Hematoxylin-eosin staining results of the complete hydatidiform mole. (B) Ki-67 expression in the complete hydatidiform mole (+). (C) p57 expression in the complete hydatidiform mole (−).

Following termination of pregnancy, pathological examination revealed hydropic villi with trophoblastic hyperplasia. Immunohistochemical analysis demonstrated negative p57 expression in villous cytotrophoblasts and a high Ki-67 proliferative index, consistent with complete hydatidiform mole (Fig. 3). Short tandem repeat (STR) analysis of the molar tissue revealed exclusively paternal alleles at all informative loci with no maternal contribution, confirming diploid androgenetic origin consistent with complete hydatidiform mole. In contrast, STR analysis of fetal tissue demonstrated biparental inheritance with alleles from both parents, confirming normal fertilization. These findings established the definitive diagnosis of CHMCF.

The patient was counseled regarding the necessity for prolonged follow-up due to the elevated risk of GTN. At the time of discharge, the patient's β-hCG level had decreased to 128,450 mIU/mL. Serial measurements demonstrated an appropriate logarithmic decline. At six weeks post-termination, β-hCG normalized to <5 mIU/mL. The patient remained asymptomatic without evidence of persistent GTN at six-month follow-up.

## 3 Discussion

CHMCF is an exceptionally rare gestational condition that poses significant diagnostic and therapeutic challenges. This case illustrates the typical presentation of CHMCF, with characteristic ultrasound findings, markedly elevated β-hCG levels, and the complex decision-making required in management [7].

The diagnosis of CHMCF relies on a combination of ultrasound findings and laboratory parameters. The characteristic honeycomb or Swiss cheese appearance of placental tissue on ultrasound, as observed in this case, reflects the hydropic degeneration of chorionic villi. However, ultrasonographic differentiation between CHMCF, singleton partial mole, and placental mesenchymal dysplasia can be challenging, particularly in early gestation [8].

Markedly elevated β-hCG levels, as seen in the present case (259,762 mIU/mL), strongly support the diagnosis of complete mole. However, definitive diagnosis requires cytogenetic analysis demonstrating diploid chromosomal content in molar tissue with a chromosomally normal coexisting fetus. When diagnostic uncertainty persists, invasive prenatal testing through amniocentesis or chorionic villus sampling for fetal karyotyping becomes a critical diagnostic tool [9].

The management of CHMCF pregnancy requires careful balancing of maternal safety against potential fetal viability. Current literature reports highly variable outcomes, with live birth rates ranging from 16 % to 40 % in cases where pregnancy continuation is attempted. However, these pregnancies carry substantial maternal risks [10].

The decision to continue or terminate a CHMCF pregnancy must be individualized, incorporating thorough consideration of maternal autonomy, available medical resources, and realistic assessment of fetal viability potential [11]. In this case, despite the patient's strong desire for pregnancy continuation, uncontrolled vaginal bleeding at 21 weeks necessitated termination to preserve maternal health. In this case, the diagnosis was conclusively established post-termination by STR analysis, which verified the androgenic origin of the mole and the biparental origin of the coexisting fetus. STR genotyping is a highly reliable method for this purpose, especially on formalin-fixed paraffin-embedded tissues, and is considered a gold standard for resolving diagnostic dilemmas in gestational trophoblastic disease.

Whether pregnancy continues to delivery or is terminated, meticulous postpartum surveillance is mandatory. The patient followed a standard protocol: weekly β-hCG measurements until three consecutive negative results (<5 mIU/mL); monthly monitoring for six months following normalization; reliable contraception during the entire surveillance period; and baseline chest imaging to exclude metastatic disease [12]. This rigorous approach enables early detection of persistent GTN, which would require prompt chemotherapy initiation.

## 4 Conclusions

CHMCF is a rare but serious obstetric condition that demands a high index of suspicion for diagnosis. Ultrasonography and markedly elevated β-hCG are key initial indicators, but definitive diagnosis often requires cytogenetic confirmation. Management must be highly individualized, involving extensive patient counseling about the significant risks of maternal complications and GTN. A multidisciplinary team approach is essential from diagnosis through post-evacuation follow-up. For patients who continue the pregnancy, vigilant monitoring is mandatory, while for all patients, meticulous post-pregnancy β-hCG surveillance is non-negotiable to mitigate the long-term risk of GTN. This case contributes to the limited literature on CHMCF and reinforces the need for standardized, multidisciplinary management protocols.

## Contributors

Yuanhua Xiang contributed to patient care, the conception of the case report and acquiring the data.

Wei Zhang contributed to acquiring and interpreting the data, and revising the article critically for important intellectual content.

Dongmin Chen contributed to acquiring and interpreting the data and drafting the manuscript.

Ting Luo contributed to acquiring the data, drafting the manuscript and revising the article critically for important intellectual content.

All authors approved the final submitted manuscript.

## Patient consent

Written informed consent was obtained from the patient for publication of the case report and accompanying images.

## Provenance and peer review

This article was not commissioned and was peer reviewed.

## Declaration of generative AI and AI-assisted technologies in the writing process

During the preparation of this work the authors used DeepSeek in order to edit grammar and enhance the readability of the manuscript. After using this tool, the authors reviewed and edited the content as needed and take full responsibility for the content of the published article.

## Funding

No specific grant from funding agencies in the public, commercial, or not-for-profit sectors supported the publication of this case report.

## Declaration of competing interest

The authors declare that they have no competing interest regarding the publication of this case report.
